# Natural Products for Drug Discovery in Cognitive Disabilities: Bibliometric Hotspots, Research Trends, Conceptual Framework, and Future Directions

**DOI:** 10.3390/ph18070983

**Published:** 2025-06-30

**Authors:** Mohammed Albratty, Maryam Halawi, Ali Mufraih Albarrati

**Affiliations:** 1 Department of Pharmaceutical Chemistry and Pharmacognosy, College of Pharmacy, Jazan University, Jazan 45142, Saudi Arabia; 2 King Salman Centre for Disability Research, Riyadh 11614, Saudi Arabia; halawima@cardiff.ac.uk; 3 Department of Haematology, College of Medicine, Cardiff University, Cardiff CF14 4XN, UK; 4 Department of Rehabilitation Sciences, College of Applied Medical Sciences, King Saud University, Riyadh 11451, Saudi Arabia; albarrati@ksu.edu.sa

**Keywords:** natural products, cognitive disabilities, bibliometric analysis, neuroprotection, emerging trends

## Abstract

**Background**: The therapeutic potential of natural products in cognitive disabilities has drawn growing attention, yet a comprehensive analysis of trends and key contributors is lacking. This study provides a bibliometric overview highlighting growth patterns, themes, and future directions. **Methods**: A comprehensive Scopus search with multistep filtering was conducted by applying keywords related to natural products and cognitive disabilities to titles, abstracts, and keywords, initially retrieving 10,011 documents. Filters for original articles and English language reduced the results to 5688. Data extracted in October 2024 were analyzed using Excel and the R-package, yielding performance and citation indices. Differential proliferation was visualized using a Sankey diagram, while thematic maps highlighted key research themes, geographic trends, and subject clusters. **Results**: The field exhibited an annual growth rate of 12.36% from 1971 to 2024, with 2021 being the most productive year (497 articles). In recent decades, citation metrics have highlighted significant impacts. Thematic maps and Sankey diagrams revealed the research focus, geographic trends, and collaboration. Alzheimer’s disease dominates the field, alongside topics such as oxidative stress, neuroprotection, and molecular docking. Emerging trends include ferroptosis, UPLC-Q-TOF-MS, and network pharmacology, which have marked advancements in therapeutic and computational approaches. **Conclusions**: This analysis underscores the dynamic and interdisciplinary nature of this field, highlighting areas for future exploration, particularly underrepresented cognitive disorders and novel therapeutic approaches.

## 1. Introduction

Cognitive disabilities refer to a broad range of conditions that affect an individual’s ability to process information, learn, communicate, and perform everyday tasks [1]. These disabilities can arise from various causes including genetic disorders, developmental abnormalities, brain injuries, and neurodegenerative diseases [2]. Common examples of cognitive impairment include autism spectrum disorder (ASD), attention deficit hyperactivity disorder (ADHD), Down syndrome, Alzheimer’s disease, dyslexia, cerebral palsy, and intellectual disabilities [3]. Cognitive disabilities can significantly affect an individual’s quality of life, limiting independence and the ability to participate fully in educational, social, and occupational settings [4]. The severity of these impairments varies widely, ranging from mild difficulties in memory or attention to profound challenges in communication and decision making [5]. Advances in neuroscience and psychology have shed light on the mechanisms underlying these conditions, enabling the development of targeted therapies and interventions [6,7]. However, many individuals with cognitive disabilities continue to face social stigma and have limited access to resources, particularly in low-resource settings [8]. Increasing awareness, promoting inclusivity, and investing in evidence-based treatments remains critical for improving outcomes [9,10]. Recent research has also explored the role of natural products, including plant-derived compounds, in the management of cognitive disabilities, offering promising avenues for therapeutic innovation through their neuroprotective and anti-inflammatory properties [5,11,12,13,14,15,16,17].

Neuropharmacology examines the impact of medications on nervous system function and the neural mechanisms that mediate their effects on behavior [18]. Neuropharmacology comprises two primary branches: behavioral and molecular [16,19]. Behavioral neuropharmacology examines the impact of pharmaceuticals on human behavior, encompassing the effects of drug dependence and addiction on the brain [20]. Molecular neuropharmacology encompasses the examination of neurons and their neurochemical interactions, aiming to create pharmaceuticals that positively influence brain function. These topics are intricately linked, as they both focus on the interactions of neurotransmitters, neuropeptides, neurohormones, neuromodulators, enzymes, second messengers, co-transporters, ion channels, and receptor proteins within the central and peripheral nervous systems [21]. Researchers are developing pharmaceuticals to address various neurological problems, including pain, neurodegenerative diseases like Parkinson’s and Alzheimer’s, psychological disorders, addiction, and others, by examining these connections [22].

Cognitive disabilities are characterized by significant limitations in intellectual functioning and adaptive behavior. According to the American Association on Intellectual and Developmental Disabilities (AAIDD), this includes: (1) an IQ below 70, (2) limitations in adaptive behaviors such as communication and self-care, and (3) onset before the age of 18 for developmental forms [23,24]. Cognitive disabilities can be developmental or acquired, and their severity may range from mild to profound. Cognitive disabilities encompass a broad range of conditions that affect intellectual functioning and adaptive behavior. These include developmental and acquired disorders such as intellectual disability (ID), Down syndrome, autism spectrum disorder (ASD), and traumatic brain injury (TBI). Specific learning disabilities like dyslexia (reading), dyscalculia (math), and dysgraphia (writing) are also recognized as forms of cognitive impairment. Other conditions include attention-deficit/hyperactivity disorder (ADHD), fetal alcohol spectrum disorders (FASD), and neurodegenerative diseases such as Alzheimer’s disease and mild cognitive impairment (MCI). Language-related disorders like aphasia, as well as genetic and developmental syndromes including fragile X syndrome, Rett syndrome, and Williams syndrome, are part of this spectrum. Additionally, developmental delay in early childhood and cerebral palsy—particularly when accompanied by cognitive deficits—are considered under the umbrella of cognitive disabilities [1,2,25,26,27].

Natural therapies are increasingly being recognized for their potential to safeguard brain structures, diminish inflammation, and enhance cognitive function in the treatment of cognitive disorders [7,14,28]. Despite increasing interest, no systematic bibliometric analysis has been performed on the advancement of research [29,30] in the intricate domain linking cognitive impairments and natural products [14,31]. In this respect, evidence highlights the relevance of focus for determining the scope and trends of studies and maps the scientific terrain. Bibliometric analysis enabled this study to quantify the number of publications, identify significant authors, assess journal influence and scope, and evaluate worldwide cooperation within the field [30,32]. Furthermore, it identifies areas of deficiency and novel elements, which will assist in the design and planning of subsequent initiatives to enhance treatment modalities [33]. The results are significant for researchers, international institutions, and grant agencies, facilitating informed decisions concerning the development of novel plant-based treatment approaches for Alzheimer’s disease, ADHD, Down syndrome, and autism spectrum disorder. This study, lacking comparable bibliometric analyses, is uniquely positioned to evaluate global research activity patterns and their relationship with clinical factors, thereby aiding in the formulation of effective strategies to enhance the quality of life of individuals with cognitive impairments.

## 2. Results

### 2.1. Annual Growth and Citation Dynamics

**Figure 1:**
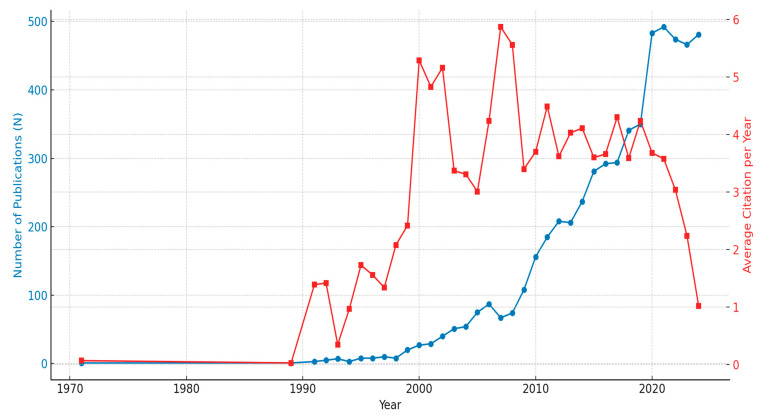
Annual growth and citation dynamics.

The bibliometric analysis of natural products and cognitive disorders from 1971 to 2024 showed a fair increase in publication output and citing dynamics (Figure 1). An impressive annual growth rate of 12.36% indicates that 5688 documents were found from 1370 sources. The average age of documents stood at 7.21 years, which indicates an increase in research activity that has not been in this volume form recently. The greatest number of original articles (data-driven studies) were published annually between the 2020s, the peak year being 2021, with 497 articles marking this decade as the most productive. Citation dynamics (Figure 1) also showed notable growth. An average document was cited 31.08 times a year (mean TCperYear). In 2007, the highest mean number of citations, no less than five a year, was reported at 5.87, followed by the totals in 2008 (5.56) and 2000 (5.29). Such a pattern indicates that some very important studies conducted in these years have had major impacts on other future studies. The earlier years, in this case the 1970s and 1980s, were characterized by very low citations, with a mean below 0.1 per year, which signifies the emergence of research in that field during those times. At the general level, this review suggests that it is a lively and rapidly developing field of research, with the greatest progress and awareness in the field being achieved in the past few decades.

### 2.2. Key Contributors in the Field

**Figure 2:**
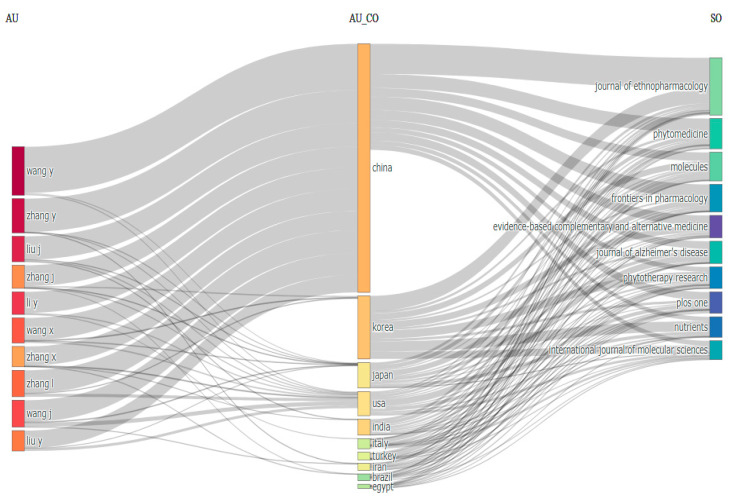
Sankey diagram. Visually representation of the connections between authors, countries, and sources. In bibliometrics, a Sankey diagram can visually represent the connections between authors, countries, and sources. Authors, countries, and sources are shown as nodes, with links indicating flows such as publications or collaborations between them.

Table 1 highlights the primary contributors to the research on natural products and cognitive disabilities. Among the authors, Zengin, G. stands out as the most productive with 42 publications, followed by Oh, M.S. (28), Choi, J.S. (27), and Shin, D.H. (26). The leading affiliations include the Ministry of Education of the People’s Republic of China (166 publications), Kyung Hee University (133), Chinese Academy of Sciences (95), and Seoul National University (65). In terms of country contributions, China leads with 1270 publications, followed by India (777), the United States (700), and South Korea (627). The most productive sources were the Journal of Ethnopharmacology (187 publications), Molecules (131), Phytomedicine (91), and Frontiers in Pharmacology (75). Figure 2 provides additional insights through a Sankey diagram that visually represents the connections between authors, countries, and sources. In bibliometrics, a Sankey diagram visually represents the connections between authors, countries, and sources. Authors, countries, and sources are shown as nodes, with links indicating flows, such as publications or collaborations between them. This visualization helps analyze patterns of collaboration, publication distribution, and information flow in the research data. The key contributors shift when focusing on the most relevant sources. Wang, Y., Zhang, Y., Liu, J., and Zhang, J. emerged as the top authors, while China, Korea, Japan, the USA, India, and Italy remained the leading countries. The key journals in this refined analysis were consistent, including the Journal of Ethnopharmacology, Phytomedicine, Molecules, and Frontiers in Pharmacology. The discrepancies between Table 1 and Figure 2 are due to the narrowed focus in Figure 2 on the most relevant sources. For instance, while Zengin, G. was the most productive author overall (Table 1), Wang, Y. was identified as the most prolific author when limited to highly relevant publications (Figure 2). This difference highlights the variability in the productivity metrics based on source selection.

### 2.3. Top-Cited and Collaborative Countries

**Figure 3:**
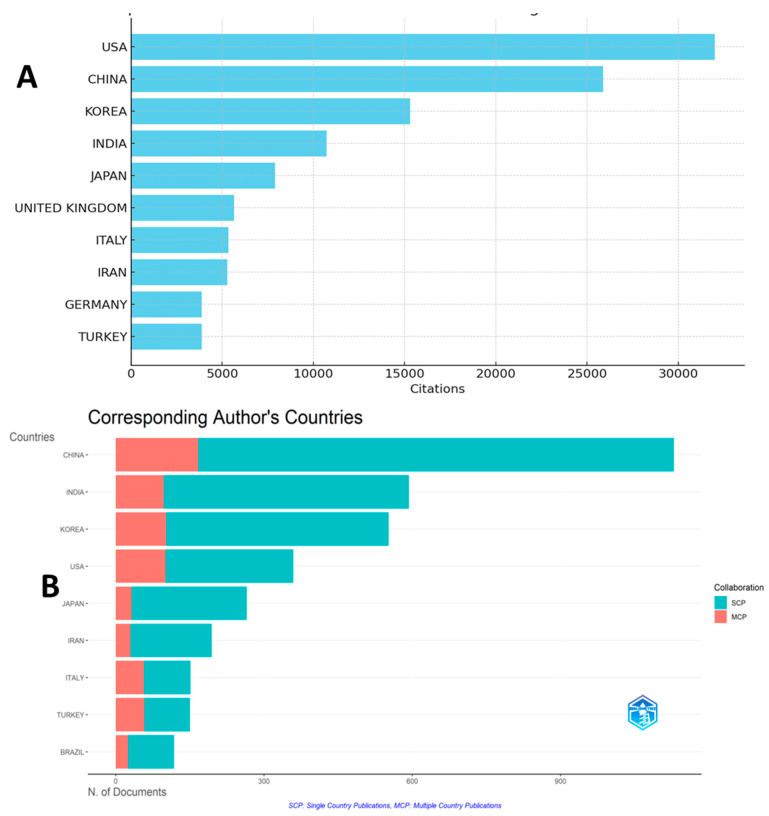
Top-cited (**A**) and collaborative (**B**) countries.

Figure 3 provides insights into the top-cited (Figure 3A) and most collaborative (Figure 3B) countries in terms of research on natural products and cognitive disabilities. In terms of total citations (TC), the USA had 32,010 citations, followed by China (25,884), Korea (15,298), and India (10,730). Other notable contributors included Japan (7901), the United Kingdom (5659), Italy (5339), Iran (5274), Germany (3881), and Turkey (3873). These metrics highlight the significant influence of the research outputs from these countries in advancing the field. Collaboration metrics show that the percentage of international co-authorships is 25.91%, reflecting a strong global research network. Publications were categorized into single-country publications (SCP) and multiple-country publications (MCP), with the MCP ratio indicating the extent of international collaboration. Among the most collaborative countries, Turkey (MCP ratio: 0.387) and Italy (0.377) demonstrated the highest levels of international partnerships, followed by the USA (0.281). On the other hand, countries such as China and India, despite their large output (1129 and 593 articles, respectively), exhibited relatively lower MCP ratios (0.148 and 0.164), indicating a higher prevalence of domestic research activities. These findings reveal a dual landscape in which countries like the USA and China dominate in citations and output, while smaller players such as Turkey and Italy excel in fostering international collaboration. This balance underscores the importance of both citation impact and collaborative effort in shaping global research.

### 2.4. Authors’ Keyword Co-Occurrence Analysis

**Figure 4:**
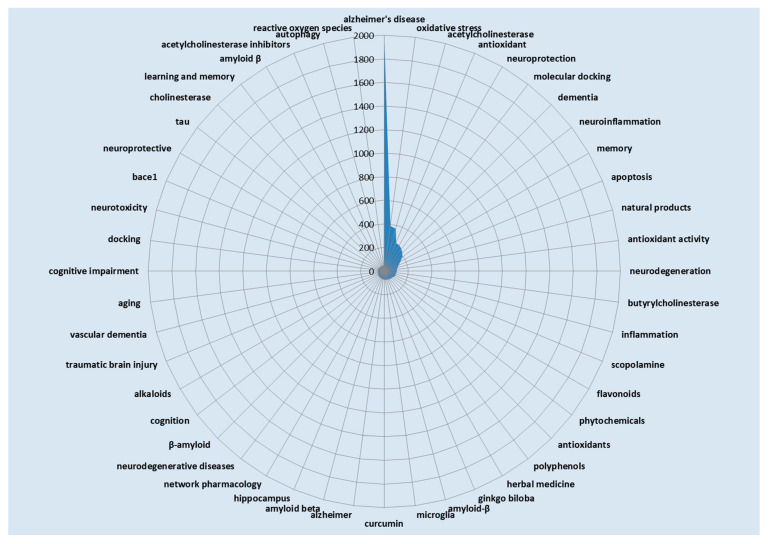
Authors’ keyword co-occurrence analysis.

Authors’ keyword co-occurrence analysis (Figure 4) identifies major research themes, emerging trends, interdisciplinary links, and research gaps, offering insights into topic relationships and guiding future studies through visualized research networks. The most frequently occurring terms in research on natural products and cognitive disabilities are the key areas of focus. Alzheimer’s disease dominates with 1935 mentions, reflecting its significance as the primary research topic in this field. Other prominent terms include oxidative stress (387), acetylcholinesterase (372), antioxidants (258), and neuroprotection (253), emphasizing the importance of understanding oxidative damage and neuroprotective mechanisms. Emerging methodologies such as molecular docking (236) underscore the integration of computational tools for drug discovery. Conditions such as dementia (215) and processes such as neuroinflammation (190) and apoptosis (130) further demonstrate the breadth of research topics exploring the underlying pathologies of cognitive impairment. Natural compounds and their bioactivities are represented by terms such as natural products (115), antioxidant activity (106), and flavonoids (94), highlighting the need for plant-based therapies. Additionally, specific enzymes, such as butyrylcholinesterase (100), and models, such as scopolamine (98), suggest targeted biochemical approaches in therapeutic development. This term analysis revealed a strong focus on neurodegenerative diseases, oxidative mechanisms, and natural product-based interventions, reflecting the interdisciplinary and translational nature of research in this field.

### 2.5. Progression of Themes in Research

**Figure 5:**
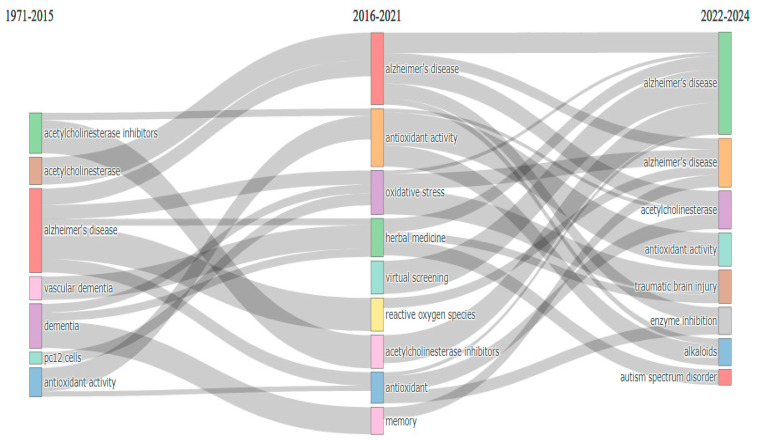
Thematic evolution of the research on natural products and cognitive disabilities.

Figure 5 illustrates the thematic evolution of research in the field of natural products and cognitive disabilities, from 1971 to 2024. During the early period (1971–2015), the focus was predominantly on acetylcholinesterase, acetylcholinesterase inhibitors, antioxidant activity, Alzheimer’s disease, dementia, oxidative stress, and related mechanisms, indicating a foundational exploration of neurochemical pathways and degenerative brain conditions. In the subsequent phase (2016–2021), research themes transitioned towards integrating broader therapeutic approaches. Alzheimer’s disease remains a dominant topic alongside emerging interests in antioxidant activity, oxidative stress, herbal medicine, and reactive oxygen species. This phase is characterized by the evolution of natural products to deal with cognitive impairments, for example, their antioxidative and neuroprotective roles. Over the past two years (2022–2024), there is evidence that further diversification has occurred within central themes. Other than Alzheimer’s disease, which remained the predominant issue, more emphasis was placed on enzyme inhibition, alkaloids, and traumatic head injuries. Themes such as autism spectrum disorder and virtual screening seem to point to a widening focus on how natural products may be applied to other cognitive disabilities, as well as drug design through computational methods. This development suggests that this is a vibrant area of research in which research on neurochemistry has expanded to encompass many aspects in relation to other cognitive disorders with a strong focus on the use of natural products. The new direction in this area of research also shows that there could be brighter prospects for addressing intricate brain disorder problems.

### 2.6. Conceptual Structure

Authors’ keywords were used because they directly reflect the researchers’ intended focus and terminology. They are essential for identifying thematic trends, keyword co-occurrence, and emerging topics, making them well-suited for bibliometric mapping and conceptual analysis. Thematic map analysis, conducted using the R package, categorized research themes in natural products and cognitive disabilities based on their centrality (importance) and density (development). Basic themes, such as acetylcholinesterase, molecular docking, antioxidant activity, natural products, and virtual screening form the foundational knowledge essential for understanding therapeutic mechanisms and advancing drug discovery. Motor themes, which are well developed and central to the field, include Alzheimer’s disease, oxidative stress, neuroprotection, neuroinflammation, apoptosis, and amyloid-β, focusing on neurodegenerative processes and therapeutic interventions. Additionally, memory and dementia-related topics, including scopolamine, acetylcholine, cognitive function, and traditional Chinese medicine, highlight experimental approaches and established therapies, such as donepezil. A niche theme, represented by Amaryllidaceae, explores specialized plant-based bioactive compounds, reflecting focused but isolated research. In contrast, declining or emerging themes such as herbal medicine, *Ginkgo biloba*, vascular dementia, and mild cognitive impairment represent areas transitioning in relevance or being integrated into broader topics. The analysis underscores the dominance of motor themes, such as neurodegeneration and Alzheimer’s disease, foundational insights from basic themes, and the potential for exploring niche areas, offering a roadmap for future research directions and highlighting critical areas requiring further development.

### 2.7. Emerging Topics

Table 2 highlights emerging topics in research on natural products and cognitive disabilities, showing their frequency and temporal progression. Recent advancements have resulted in a growing interest in cutting-edge methodologies and evolving therapeutic approaches. Key emerging technologies include UPLC-Q-TOF-MS (first quartile [Q1] 2023, median 2024), ferroptosis (Q1 2022, median 2024), and network pharmacology (Q1 2021, median 2022), all of which emphasize the application of advanced analytical and computational tools to unravel complex biological mechanisms. Molecular dynamics simulation (Q1 2021, median 2023) and molecular docking (Q1 2020, median 2022) demonstrated an increasing reliance on computational modeling for understanding drug–target interactions. Topics such as absorption, distribution, metabolism, excretion, and toxicity (Q1 2021, median 2023) and MD simulations further underscore the emphasis on computational pharmacology in optimizing drug discovery. Emerging biological processes, including ferroptosis, autophagy (Q1 2019, median 2022), and neuroinflammation (Q1 2018, median 2021), indicate a focus on novel pathological pathways in cognitive disorders. Long-standing yet continually evolving topics, such as antioxidant activity, oxidative stress, and phytochemicals, have maintained their relevance, bridging traditional and modern therapeutic approaches. Among the diseases, Alzheimer’s disease is the most frequently studied emerging topic, with consistent growth (Q1 2019, median 2021), reflecting its significance in neurodegenerative research. These trends highlight the interdisciplinary nature of this field, which combines natural products, computational approaches, and advanced methodologies to address cognitive disabilities.

## 3. Discussion

Research on natural products and cognitive illnesses from 1971 to 2024 demonstrates a dynamic and rapidly evolving field characterized by an impressive annual growth rate of 12.36%. The publication of 5688 papers from 1370 sources indicates a consistent increase in research output, underscoring the global recognition of natural products as a valuable area of study. The peak year of 2021, with 497 articles, indicates heightened interest, which may be driven by advancements in technology, such as molecular docking [16,17], and an increasing understanding of plant-based therapies for neurological disorders [9,34,35]. The escalation in citation dynamics underscores the significance of fundamental research as it has influenced subsequent studies and positioned natural products [9,34,35] as a cornerstone of innovation in therapeutic development. These results complement earlier research, stressing the need for natural products to treat several diseases, including cancer [27], cardiovascular diseases [36], diabetes [37], and neurological diseases such as Alzheimer’s disease [2,10,34]. Although countries like China and India produce a high volume of research, they tend to have lower levels of international collaboration—possibly due to robust domestic research networks, language preferences, or national funding priorities—underscoring the importance of encouraging more global partnerships in future studies.

This work supports the importance of natural chemicals in medication development, especially their bioactive characteristics that support antioxidant, anti-inflammatory, and neuroprotective activities. Through trend mapping and research impact analysis, this study supports the increasing agreement that natural products are viable strategies for controlling complicated disorders and for developing therapeutic treatments worldwide.

This research indicates that China is the leading country in terms of output in the field of natural products and cognitive disabilities more probably under the influence that traditional Chinese medicine (TCM) plays in this area. TCM has been known for a long time to combine natural products and holistic approaches for the treatment of neurological and cognitive dysfunctions, which to some extent explains the high volume of research activities in China [15,31,38]. Newer research has drawn attention to the use of traditional medicine in modern paradigms for the treatment of cognitive disabilities, stressing its place in rehabilitation and treatment [15]. TCM has been shown to be effective in the management of cognitive deficits following stroke. Combining TCM with new practices such as virtual reality (VR) can help enhance memory and executive function recovery in stroke survivors [26]. Additionally, there have been developments in neuroprosthetics and brain–computer interfaces that reportedly show better results in cognitive rehabilitation and are said to be based on TCM [39]. Another traditional approach that has been applied in a number of post-stroke patients is music therapy, which has been used in the treatment of emotional and cognitive rehabilitation [40]. Newer techniques, such as dynamic brain network mapping and deep brain stimulation (DBS), also advocate the combination of traditional techniques with new methods for the treatment of neurological and cognitive disorders. The incorporation of traditional medicine into holistic approaches, such as the biopsychosocial model, has effective implications in steadying the complete recovery of older populations from both mental and physical aspects [16,27,36]. Understanding the role of TCM and other traditional medicines is essential in developing methods for cognitive rehabilitation and dealing with highly complex neurological disorders [15].

Zengin, G., a leading scholar in natural product research, has significantly advanced the understanding of plant-based therapies for managing chronic conditions such as diabetes, Alzheimer’s disease, and oxidative stress. His work emphasizes phytochemical profiling, enzyme inhibition, and antioxidant activity using both in vitro and in silico methodologies. Notable studies include the evaluation of *Salvia syriaca* L. for its antidiabetic, anti-Alzheimer’s, and antioxidant properties [28] and *Ajuga chamaecistus* for its neuroprotective and skin-care applications [41]. Zengin’s research on mangiferin and essential oils from *Sideritis galatica* demonstrated their inhibitory effects on enzymes associated with Alzheimer’s disease and diabetes, thus providing molecular insights into their therapeutic roles [16,37]. Using advanced techniques, such as HPLC-MS/MS, Zengin profiled phenolic compounds in species such as *Ferula halophila* and *Scrophularia lucida*, combining these analyses with antioxidant, anti-inflammatory, and docking studies to explore their multifunctional applications [42]. Comparative studies of Apiaceae species have highlighted their antioxidant, antimicrobial, and cytotoxic properties, emphasizing their potential against cancer [43]. By integrating cutting-edge analytical approaches with pharmacological evaluations, Zengin bridges phytochemistry and therapeutic innovation, offering diverse applications for natural products in combating chronic diseases and oxidative stress-related conditions [28,37,41,42,43,44,45,46,47,48].

This conceptual shift has focused research after 2022 on antioxidants and their use to regulate cholinesterase, which plays a role in the pathology of Alzheimer’s, according to the analysis of the study’s objectives. Alzheimer’s disease, the most prevalent form of dementia, degrades brain functions over time. As the condition advances, acetylcholine levels in aged brains decrease, causing cognitive decline. Acetylcholinesterase inhibitors (AChEIs) increase synaptic acetylcholine levels and restore cholinergic neurotransmission [2,10,31,34]. Current drugs may not sufficiently modulate acetylcholine levels to provide a complete therapeutic response, and selective cholinesterase inhibitors without dose-limiting adverse effects are unavailable [49]. Synthetic drugs such as tacrine, donepezil, and rivastigmine can also induce gastrointestinal and bioavailability issues. Thus, the search for more effective AChEIs, especially from natural sources, is ongoing. Reactive oxygen species (ROS) generate oxidative stress, which oxidizes biomolecules and damages cells, thereby causing aging. Recently, plant antioxidants have garnered attention because they reduce oxidative damage and may prevent aging and neurological illnesses.

Neuroprotection has become one of the central motor themes in research concerning natural compounds with respect to the degenerative diseases such as Alzheimer’s disease [50]. The natural compound extracts provided in these studies tend to have functional properties, including the ability to manage oxidative stress, inhibit β-amyloid (Aβ) aggregation, and modulate inflammatory pathways [2,6,10,34,51]. These compounds can reduce age-related neurodegeneration. Such work basically extends the findings of Bastianetto et al., who demonstrated that catechin gallates from extracts of green and black tea-infused cultures could protect cells from the Aβ-induced toxicity by inhibiting the apoptotic events and aggregation of Aβ [34]. Similarly, Chu et al. confirmed that treatment with Ginsenoside Rg5 isolated from Panax ginseng had remedial effects on cognitive deficits in rats by reducing neuroinflammation and Aβ deposition and increasing acetylcholine levels [2]. *Bacopa monnieri* has been extensively studied for its anti-nutritional properties. It was found to be protective against Aβ-induced neurotoxicity and to improve cognitive function due to its antioxidant and acetylcholinesterase inhibitory properties [52]. In addition, beta-asarone from *Acorus tatarinowii* was found to prevent cognitive impairment and neuronal apoptosis in Aβ-injected rats through the reversal of caspase activation and JNK phosphorylation processes [10]. These studies highlight that neuroprotection is an area worthy of focus in research by creating strategies that are based on both modern and traditional wisdom concerning natural products through the use of antioxidant activity, anti-inflammatory activity, and Aβ aggregation inhibition to solve cognitive decline.

A bibliometric analysis of trends in UPLC-Q-TOF-MS usage shows its growing relevance as a revolutionary technique for the study of the application of natural products in the treatment of cognitive dysfunction. In addition, this technique of analysis is the most useful in identifying and explaining the mechanism of action of bioactive compounds in the case of neurodegenerative diseases, which include but are not restricted to Alzheimer’s disease. Recent studies have further bolstered their relevance. Qiu et al. performed UPLC-Q-TOF-MS on *Aurantii Fructus* and *Aurantii Fructus Immaturus* and reported the discovery of 50 compounds with neuroprotective effects categorized as Alzheimer’s, NF-κB signaling, and apoptosis pathways [53]. Wang et al. investigated the anti-Alzheimer’s properties of Mume Flos using UHPLC-Q-Orbitrap-MS/MS and noted that rutin and chlorogenic acid were inferior to others in terms of their efficacy in the inhibition of Aβ deposition in *Caenorhabditis elegans* models [54]. Similarly, Zhang et al. used high-resolution LC-Q-TOF-MS to demonstrate the significant microinhibitory activity of nitidine from *Zanthoxylum nitidum* as a strong candidate for acetylcholinesterase activity inhibition [55]. These findings illustrate the potential power of UPLC-Q-TOF-MS techniques for the development and application of natural product-derived medicine against cognitive disorders, and highlight the possible advancements that such techniques would herald for the integration of herbal research and contemporary pharmacology.

Bibliometric analysis showed that research on the processes of regulation of cell death, such as ferroptosis, has been a hot topic in the areas of cognitive disorders; however, recent studies have highlighted the importance of these processes in Alzheimer’s disease and several other degenerative diseases. Gong et al. showed that curculigoside significantly restrained the progression of Alzheimer’s disease and ameliorated cognitive dysfunction by upregulating GPX4 and inhibiting the SLC7A11 pathway [31]. A similar effort was made by Li et al., who investigated avicularin as a means to ameliorate cognitive defects associated with Alzheimer’s disease by modifying the NOX4/Nrf2 axis in PC12 cell lines and transgenic models of AD [17]. Long et al. investigated the neuronal protection offered by the SuanZaoRen decoction and its ability to inhibit Alzheimer’s disease-mediated neuronal loss and improve synaptic connectivity by targeting the DJ-1/Nrf2 signaling pathway [16]. Tao et al. evaluated the biochemical and physiological effects of oleanonic acid on ferroptosis and mitochondrial damage via Nrf2/HO-1 signaling [14]. More recently, Yong et al. characterized Thonningianin A, a new compound that inhibits ferroptosis and described its use in Alzheimer’s disease therapy via GPX4 stimulation through the AMPK/Nrf2 pathway [12]. Moreover, *Penthorum chinense* Pursh (PEF) has been shown to inhibit ferroptosis in Alzheimer’s disease cellular and C. elegans models by targeting oxidative stress and lipid peroxidation processes [11]. These studies confirm the bibliometric findings by showcasing ferroptosis as a key factor in neurodegenerative diseases, and natural products have been proposed as potential anti-ferroptotic agents to improve cognition. While emerging topics such as ferroptosis and UPLC-Q-TOF-MS signal promising directions, their clinical translation remains limited by challenges in validation, including biological complexity, lack of standardized protocols, and the need for robust in vivo and clinical evidence to support their therapeutic feasibility.

## 4. Limitations

Scopus- and English-exclusive databases have constraints. Examples of these problems include inadequate citation coverage, publishing bias, language and geographical discrepancies, subject limitations, and data-input inaccuracies. Researchers ought to employ several databases, examine scholarly papers in different languages, engage in interdisciplinary collaboration, and investigate supplementary publications to mitigate these limitations. This guarantees a comprehensive and reliable research environment.

## 5. Materials and Methods

### 5.1. Search Strategy and Data Extraction

A comprehensive and multistep search was conducted in the Scopus database to identify relevant literature on the use of natural products to address cognitive disabilities and related conditions. The search terms included keywords related to natural products, such as “phytotherapy”, “herbal medicines”, “plant extract”, “natural product”, “natural compound”, “natural molecule”, “phytochemical”, “secondary metabolite”, “bioactive compounds”, “biologically active compounds”, “phytonutrient”, “plant-derived chemical”, and “plant-derived compound,” combined with terms associated with cognitive disabilities and related conditions, including “degenerative brain disease”, “cognitive disability”, “dementia”, “autism spectrum disorder”, “ADHD”, “Down syndrome”, “fragile X syndrome”, “traumatic brain injury”, “Alzheimer’s disease”, “cerebral palsy”, “aphasia”, “dyslexia”, and “intellectual disabilities”. These keywords were extracted from an extensive search in the related literature [1,7,17,19,27,54]. To ensure the quality and relevance of the data, specific filters were applied during the search process. Only original research articles were included, and the language was restricted to English. These filters were applied after the initial retrieval of 10,011 documents, resulting in a refined dataset of 5688 articles suitable for bibliometric analysis. This approach ensured the inclusion of peer-reviewed, research-focused studies relevant to natural products and cognitive disabilities. The search results were exported in the CSV and BibTeX formats, and the data were extracted in October 2024.

### 5.2. Analysis and Visualizations

Annual trends were examined using Microsoft Excel, while bibliometric statistics and visualizations were produced using the R-package software Version 5.0.1 [55], enabling comprehensive mapping of research focus points, geographical research regions, and subject clusters. The package measures the intensity of the linkages or connections among authors, institutions, and keywords inside a network map. The thematic map depicts the primary issues in the field according to the author keywords. The objective of constructing a thematic map was to understand the present conditions and assess the future direction of research progress on the subject. The map depicts the strength of internal density, highlighting inter-cluster development and external connections, and underscoring the significance of the study to a specific focal location. It is partitioned into four quadrants. The term thematic map classifies the principal study themes and subjects within a certain domain [55,56].

## 6. Conclusions

This study shows that research on natural products and cognitive disabilities is rapidly evolving, with a notable surge in global interest and collaboration. The field has been growing at an annual rate of 12.36%, with a sharp rise in citations since 2021, reflecting its increasing relevance. Major contributions from countries like China, the USA, and South Korea have helped shape this progress. The research landscape has expanded from early focus areas—such as oxidative stress and acetylcholinesterase inhibition—to newer themes like ferroptosis and advanced computational tools, underscoring the field’s interdisciplinary growth.

Despite this progress, several gaps remain. Alzheimer’s disease continues to dominate the research spotlight, often overshadowing other important cognitive conditions like autism spectrum disorders and traumatic brain injuries. Additionally, many potentially valuable bioactive compounds from less-studied plant species remain underexplored. Topics such as vascular dementia, which once received more attention, are now on the decline and require renewed scientific focus. Promising innovations, including UPLC-Q-TOF-MS and studies on ferroptosis, show potential but still need validation through clinical trials.

To move the field forward, researchers are encouraged to broaden their focus to include a wider range of cognitive disorders, enhance international collaboration to share resources and expertise, and utilize emerging technologies to uncover new therapeutic pathways. Emphasizing translational research and improving data sharing will also help bridge the gap between lab-based discoveries and real-world clinical impact. By addressing these challenges while continuing to build on core strengths, the field can make meaningful advances in the development of therapies for cognitive disabilities.

## Figures and Tables

**Table 1:** Key contributors in the field.

Authors	N	Affiliation	N	Sources	N	Country	N
Zengin, G.	42	Ministry of Education of the People’s Republic of China	166	Journal of Ethnopharmacology	187	China	1270
Oh, M.S.	28	Kyung Hee University	133	Molecules	131	India	777
Choi, J.S.	27	Chinese Academy of Sciences	95	Phytomedicine	91	United States	700
Shin, D.H.	26	Seoul National University	65	Frontiers in Pharmacology	75	South Korea	627
Ryu, J.H.	23	Shenyang Pharmaceutical University	62	International Journal of Molecular Sciences	75	Japan	389
Oboh, G.	22	King Saud University	58	Journal of Alzheimer’s Disease	75	Italy	240
Hritcu, L.	21	Chinese Academy of Medical Sciences & Peking Union Medical College	55	Phytotherapy Research	74	Iran	235
Jung, H.A.	21	Selçuk Üniversitesi	52	Nutrients	71	Turkey	214
Bi, K.	20	National Research Centre	51	Evidence-Based Complementary and Alternative Medicine	69	Saudi Arabia	199
Choi, S.J.	20	King Abdulaziz University	50	PLOS One	68	Germany	197

N: number of documents.

**Table 2:** Emerging topics.

Item	Frequency	Year_q1	Year_med	Year_q3
UPLC-Q-TOF-MS	6	2023	2024	2024
Ferroptosis	9	2022	2024	2024
Network pharmacology	65	2021	2022	2024
Molecular dynamics simulation	28	2021	2023	2024
ADMET	18	2021	2023	2024
Molecular docking	236	2020	2022	2023
Alzheimer’s disease	628	2019	2021	2023
Phytochemicals	85	2019	2021	2023
Autophagy	48	2019	2022	2023
Neuroinflammation	190	2018	2021	2023
Neurodegeneration	106	2016	2020	2022
Antioxidant	258	2015	2019	2022
Antioxidant activity	106	2015	2019	2022
Flavonoids	94	2015	2020	2022
Oxidative stress	387	2014	2019	2022

Abbreviations: med: median; UPLC-Q-TOF-MS: ultra-performance liquid chromatography quadrupole time-of-flight mass spectrometry; ADMET: absorption, distribution, metabolism, excretion, and toxicity.

## Data Availability

The original contributions presented in this study are included in the article. Further inquiries can be directed to the corresponding author.

## References

[1] Uabundit N., Wattanathorn J., Mucimapura S., Ingkaninan K. (2010). Cognitive enhancement and neuroprotective effects of *Bacopa monnieri* in Alzheimer’s disease model. J. Ethnopharmacol..

[2] Chu S., Gu J., Feng L., Liu J., Zhang M., Jia X., Liu M., Yao D. (2014). Ginsenoside Rg5 improves cognitive dysfunction and beta-amyloid deposition in STZ-induced memory impaired rats via attenuating neuroinflammatory responses. Int. Immunopharmacol..

[3] Sigafoos J., Roche L., O’Reilly M.F., Lancioni G.E. (2021). Persistence of primitive reflexes in developmental disorders. Curr. Dev. Disord. Rep..

[4] Yeung P., Breheny M. (2021). Quality of life among older people with a disability: The role of purpose in life and capabilities. Disabil. Rehabil..

[5] Megari K., Thomaidou E., Chatzidimitriou E. (2024). Highlighting the Neuropsychological Consequences of COVID-19: Evidence From a Narrative Review. INQUIRY J. Health Care Organ. Provis. Financ..

[6] Lim G.P., Chu T., Yang F., Beech W., Frautschy S.A., Cole G.M. (2001). The curry spice curcumin reduces oxidative damage and amyloid pathology in an Alzheimer transgenic mouse. J. Neurosci..

[7] Park C.H., Choi S.H., Koo J.W., Seo J.H., Kim H.S., Jeong S.J., Suh Y.H. (2002). Novel cognitive improving and neuroprotective activities of *Polygala tenuifolia* willdenow extract, BT-11. J. Neurosci. Res..

[8] Hepperlen R.A., Biggs J., Mwandileya W., Rabaey P., Ngulube E., Hearst M.O. (2021). Using community-based interventions to reduce public stigma of children with disabilities: A feasibility study. J. Appl. Res. Intellect. Disabil..

[9] Budzynska B., Boguszewska-Czubara A., Kruk-Slomka M., Skalicka-Wozniak K., Michalak A., Musik I., Biala G. (2015). Effects of imperatorin on scopolamine-induced cognitive impairment and oxidative stress in mice. Psychopharmacology.

[10] Geng Y., Li C., Liu J., Xing G., Zhou L., Dong M., Li X., Niu Y. (2010). Beta-asarone improves cognitive function by suppressing neuronal apoptosis in the beta-amyloid hippocampus injection rats. Biol. Pharm. Bull..

[11] Yong Y.Y., Yan L., Wang B.D., Fan D.S., Guo M.S., Yu L., Wu J.M., Qin D.L., Law B.Y.K., Wong V.K.W. (2024). *Penthorum chinense* Pursh inhibits ferroptosis in cellular and *Caenorhabditis elegans* models of Alzheimer’s disease. Phytomedicine.

[12] Yong Y., Yan L., Wei J., Feng C., Yu L., Wu J., Guo M., Fan D., Yu C., Qin D. (2024). A novel ferroptosis inhibitor, Thonningianin A, improves Alzheimer’s disease by activating GPX4. Theranostics.

[13] Tao L., Liu Z., Li X., Wang H., Wang Y., Zhou D., Zhang H. (2024). Oleanonic acid ameliorates mutant Aβ precursor protein-induced oxidative stress, autophagy deficits, ferroptosis, mitochondrial damage, and ER stress in vitro. Biochim. Biophys. Acta Mol. Basis Dis..

[14] Tang H., He K., Zhao K., Zheng C., Wu W., Jin W., Yang L., Xie B. (2024). Protective Effects of Hinokitiol on Neuronal Ferroptosis by Activating the Keap1/Nrf2/HO-1 Pathway in Traumatic Brain Injury. J. Neurotrauma.

[15] Qiu M., Zhang J., Wei W., Zhang Y., Li M., Bai Y., Wang H., Meng Q., Guo D.A. (2024). Integrated UPLC/Q-TOF-MS/MS Analysis and Network Pharmacology to Reveal the Neuroprotective Mechanisms and Potential Pharmacological Ingredients of Aurantii Fructus Immaturus and Aurantii Fructus. Pharmaceuticals.

[16] Long Q., Li T., Zhu Q., He L., Zhao B. (2024). SuanZaoRen decoction alleviates neuronal loss, synaptic damage and ferroptosis of AD via activating DJ-1/Nrf2 signaling pathway. J. Ethnopharmacol..

[17] Li Z., Lu Y., Zhen Y., Jin W., Ma X., Yuan Z., Liu B., Zhou X.L., Zhang L. (2024). Avicularin inhibits ferroptosis and improves cognitive impairments in Alzheimer’s disease by modulating the NOX4/Nrf2 axis. Phytomedicine.

[18] Mishra V.H., Khade A., Noman O. (2025). Drug effects on the nervous system: Mechanisms and future directions in neuropharmacological therapy. Adv. Hum. Biol..

[19] Um M.Y., Choi W.H., Aan J.Y., Kim S.R., Ha T.Y. (2006). Protective effect of Polygonum multiflorum Thunb on amyloid β-peptide 25-35 induced cognitive deficits in mice. J. Ethnopharmacol..

[20] Ghallab Y.K., Elassal O.S. (2024). Biochemical and Neuropharmacology of Psychiatric Disorders. Nutrition and Psychiatric Disorders: An Evidence-Based Approach to Understanding the Diet-Brain Connection.

[21] Orchard I., Leyria J., Al-Dailami A., Lange A.B. (2021). Fluid secretion by Malpighian tubules of Rhodnius prolixus: Neuroendocrine control with new insights from a transcriptome analysis. Front. Endocrinol..

[22] Oyebanjo O.T., Adetuyi B.O., Adeoye A.D., Adetuyi O.A., Oni P.G., Ogunlana O.O. (2024). Neuropharmacology and neurotherapeutics: Advancing the understanding and treatment of neurological disorders. Biochemical and Molecular Pharmacology in Drug Discovery.

[23] Tassé M.J., Luckasson R., Schalock R.L. (2016). The relation between intellectual functioning and adaptive behavior in the diagnosis of intellectual disability. Intellect. Dev. Disabil..

[24] Tassé M.J., Grover M. (2021). American association on intellectual and developmental disabilities (aaidd). Encyclopedia of Autism Spectrum Disorders.

[25] Boat T.F., Wu J.T., Committee to Evaluate the Supplemental Security Income Disability Program for Children with Mental Disorders, Board on the Health of Select Populations, Board on Children, Youth, and Families, Institute of Medicine, The National Academies of Sciences, Engineering, and Medicine (2015). Clinical characteristics of intellectual disabilities. Mental Disorders and Disabilities Among Low-Income Children.

[26] Bao X., Song X., Deng H., Jiang L. (2024). The Impact of Virtual Reality Training Combined with Traditional Chinese Medicine Health Preservation Therapy on Cognitive Function, Neurological Function, and Physical Function of Stroke Patients. Int. J. Neurosci..

[27] Li L., Li W.J., Zheng X.R., Liu Q.L., Du Q., Lai Y.J., Liu S.Q. (2022). Eriodictyol ameliorates cognitive dysfunction in APP/PS1 mice by inhibiting ferroptosis via vitamin D receptor-mediated Nrf2 activation. Mol. Med..

[28] Bahadori M.B., Dinparast L., Zengin G., Sarikurkcu C., Bahadori S., Asghari B., Movahhedin N. (2017). Functional components, antidiabetic, anti-Alzheimer’s disease, and antioxidant activities of *Salvia syriaca* L.. Int. J. Food Prop..

[29] Abdelwahab S.I., Taha M.M.E., Mariod A.A. (2025). Performance analysis, conceptual mapping, and emerging trends for Gum Arabic research: A comprehensive bibliometric analysis from 1916 to 2023. Food Prod. Process. Nutr..

[30] Abdelwahab S.I., Farasani A., Alfaifi H.A., Hassan W. (2024). Trends and dynamics in facelift surgery research: A bibliometric analysis of the top 50 most cited papers. Chin. J. Plast. Reconstr. Surg..

[31] Gong Y., Wang Y., Li Y., Weng F., Chen T., He L. (2024). Curculigoside, a traditional Chinese medicine monomer, ameliorates oxidative stress in Alzheimer’s disease mouse model via suppressing ferroptosis. Phytother. Res..

[32] Van Eck N., Waltman L. (2010). Software survey: VOSviewer, a computer program for bibliometric mapping. Scientometrics.

[33] Bérdi M. (2023). Bibliometric analysis of Hungarian-related publications in suicidal behavior research of the last three decades. Psychiatr. Hung..

[34] Bastianetto S., Yao Z.X., Papadopoulos V., Quirion R. (2006). Neuroprotective effects of green and black teas and their catechin gallate esters against β-amyloid-induced toxicity. Eur. J. Neurosci..

[35] Das A., Shanker G., Nath C., Pal R., Singh S., Singh H.K. (2002). A comparative study in rodents of standardized extracts of *Bacopa monniera* and *Ginkgo biloba*—Anticholinesterase and cognitive enhancing activities. Pharmacol. Biochem. Behav..

[36] Hybertson B.M., Gao B., Bose S.K., McCord J.M. (2011). Oxidative stress in health and disease: The therapeutic potential of Nrf2 activation. Mol. Asp. Med..

[37] Picot M.C.N., Zengin G., Mollica A., Stefanucci A., Carradori S., Mahomoodally M.F. (2017). In vitro and in silico studies of mangiferin from aphloia theiformis on key enzymes linked to diabetes type 2 and associated complications. Med. Chem..

[38] Wang X., Zhao H., Wu M., Gan Y., Zhang S., Wu D., Ou J., Jin C., Zhang W. (2024). Integrated UHPLC-Q-Orbitrap-MS/MS Method and Network Pharmacology for Exploring the Active Components and Potential Mechanisms of Neuroprotective Effect of the n-Butanol Part of Mume Flos. Nat. Prod. Commun..

[39] Tao Q., Chao H., Fang D., Dou D. (2024). Progress in neurorehabilitation research and the support by the National Natural Science Foundation of China from 2010 to 2022. Neural Regen. Res..

[40] Lin F., Huang D., He N., Gu Y., Wu Y. (2017). Effect of music therapy derived from the five elements in Traditional Chinese Medicine on post-stroke depression. J. Tradit. Chin. Med..

[41] Movahhedin N., Zengin G., Bahadori M.B., Sarikurkcu C., Bahadori S., Dinparast L. (2016). *Ajuga chamaecistus* subsp. scoparia (Boiss.) Rech.f.: A new source of phytochemicals for antidiabetic, skin-care, and neuroprotective uses. Ind. Crops Prod..

[42] Zengin G., Uysal A., Diuzheva A., Gunes E., Jekő J., Cziáky Z., Picot-Allain C.M.N., Mahomoodally M.F. (2018). Characterization of phytochemical components of *Ferula halophila* extracts using HPLC-MS/MS and their pharmacological potentials: A multi-functional insight. J. Pharm. Biomed. Anal..

[43] Zengin G., Sinan K.I., Ak G., Mahomoodally M.F., Paksoy M.Y., Picot-Allain C., Glamocilja J., Sokovic M., Jekő J., Cziáky Z. (2020). Chemical profile, antioxidant, antimicrobial, enzyme inhibitory, and cytotoxicity of seven Apiaceae species from Turkey: A comparative study. Ind. Crops Prod..

[44] Savran A., Zengin G., Aktumsek A., Mocan A., Glamoćlija J., Ćirić A., Soković M. (2016). Phenolic compounds and biological effects of edible: *Rumex scutatus* and *Pseudosempervivum sempervivum*: Potential sources of natural agents with health benefits. Food Funct..

[45] Zengin G., Nithiyanantham S., Locatelli M., Ceylan R., Uysal S., Aktumsek A., Selvi P.K., Maskovic P. (2016). Screening of in vitro antioxidant and enzyme inhibitory activities of different extracts from two uninvestigated wild plants: *Centranthus longiflorus* subsp. longiflorus and Cerinthe minor subsp. auriculata. Eur. J. Integr. Med..

[46] Zengin G., Sarıkürkçü C., Aktümsek A., Ceylan R. (2015). Antioxidant potential and inhibition of key enzymes linked to Alzheimer’s diseases and diabetes mellitus by monoterpene-rich essential oil from *Sideritis galatica* Bornm. Endemic to Turkey. Rec. Nat. Prod..

[47] Zengin G., Sarikurkcu C., Gunes E., Uysal A., Ceylan R., Uysal S., Gungor H., Aktumsek A. (2015). Two Ganoderma species: Profiling of phenolic compounds by HPLC-DAD, antioxidant, antimicrobial and inhibitory activities on key enzymes linked to diabetes mellitus, Alzheimer’s disease and skin disorders. Food Funct..

[48] Zengin G., Stefanucci A., Rodrigues M.J., Mollica A., Custodio L., Aumeeruddy M.Z., Mahomoodally M.F. (2019). *Scrophularia lucida* L. as a valuable source of bioactive compounds for pharmaceutical applications: In vitro antioxidant, anti-inflammatory, enzyme inhibitory properties, in silico studies, and HPLC profiles. J. Pharm. Biomed. Anal..

[49] Mukherjee P.K., Kumar V., Mal M., Houghton P.J. (2007). Acetylcholinesterase inhibitors from plants. Phytomedicine.

[50] Heo H.J., Kim D.O., Shin S.C., Kim M.J., Kim B.G., Shin D.H. (2004). Effect of Antioxidant Flavanone, Naringenin, from *Citrus junos* on Neuroprotection. J. Agric. Food Chem..

[51] Yu M.S., Leung S.K.Y., Lai S.W., Che C.M., Zee S.Y., So K.F., Yuen W.H., Chang R.C.C. (2005). Neuroprotective effects of anti-aging oriental medicine *Lycium barbarum* against β-amyloid peptide neurotoxicity. Exp. Gerontol..

[52] Limpeanchob N., Jaipan S., Rattanakaruna S., Phrompittayarat W., Ingkaninan K. (2008). Neuroprotective effect of *Bacopa monnieri* on beta-amyloid-induced cell death in primary cortical culture. J. Ethnopharmacol..

[53] Zhang J.-N., Pei Z.-D., Wang W.-Y., Zhao M.-Y., Pei W.-H., Zhang H., Yin H.-B., Wang T.-M., Xin G.-Z., Xie M. (2024). Integration of High-Resolution LC-Q-TOF Mass Spectrometry and Multidimensional Chemical-Biological Analysis to Detect Nanomolar-Level Acetylcholinesterase Inhibitors from Different Parts of *Zanthoxylum nitidum*. J. Agric. Food Chem..

[54] McCarney R., Warner J., Iliffe S., Van Haselen R., Griffin M., Fisher P. (2007). The Hawthorne Effect: A randomised, controlled trial. BMC Med. Res. Methodol..

[55] Aria M., Cuccurullo C. (2017). bibliometrix: An R-tool for comprehensive science mapping analysis. J. Informetr..

[56] Alkhammash R. (2023). Bibliometric, network, and thematic mapping analyses of metaphor and discourse in COVID-19 publications from 2020 to 2022. Front. Psychol..

